# Multi-omics analysis of the biological mechanism of the pathogenesis of non-alcoholic fatty liver disease

**DOI:** 10.3389/fmicb.2024.1379064

**Published:** 2024-07-26

**Authors:** Jie Lin, Ruyi Zhang, Huaie Liu, Yunzhen Zhu, Ningling Dong, Qiu Qu, Hongyan Bi, Lihua Zhang, Ou Luo, Lei Sun, Mengjuan Ma, Jing You

**Affiliations:** ^1^Department of General Practice, The First Affiliated Hospital of Kunming Medical University, Kunming, China; ^2^Department of Infectious Diseases and Hepatology, The First People’s Hospital of Yunnan Province, The Affiliated Hospital of Kunming University of Science and Technology, Kunming, China; ^3^Department of Geriatric Gastroenterology, The First Affiliated Hospital of Kunming Medical University, Kunming, China; ^4^Department of Gastroenterology, The First Affiliated Hospital of Kunming Medical University, Kunming, China; ^5^Department of Health Examination, The First Affiliated Hospital of Kunming Medical University, Kunming, China

**Keywords:** non-alcoholic fatty liver disease, gut microbiota, differential metabolites, differentially expressed genes, multi-omics

## Abstract

**Background:**

Non-alcoholic fatty liver disease (NAFLD) is a type of liver metabolic syndrome. Employing multi-omics analyses encompassing the microbiome, metabolome and transcriptome is crucial for comprehensively elucidating the biological processes underlying NAFLD.

**Methods:**

Hepatic tissue, blood and fecal samples were obtained from 9 NAFLD model mice and 8 normal control mice. Total fecal microbiota DNA was extracted, and 16S rRNA was amplified, to analyze alterations in the gut microbiota (GM) induced by NAFLD. Subsequently, diagnostic strains for NAFLD were screened, and their functional aspects were examined. Differential metabolites and differentially expressed genes were also screened, followed by enrichment analysis. Correlations between the differential microbiota and metabolites, as well as between the DEGs and differential metabolites were studied. A collinear network involving key genes-, microbiota-and metabolites was constructed.

**Results:**

*Ileibacterium* and *Ruminococcaceae*, both belonging to Firmicutes; *Olsenella*, *Duncaniella* and *Paramuribaculum* from Bacteroidota; and *Bifidobacterium*, *Coriobacteriaceae_UCG_002* and *Olsenella* from Actinobacteriota were identified as characteristic strains associated with NAFLD. Additionally, differentially expressed metabolites were predominantly enriched in tryptophan, linoleic acid and methylhistidine metabolism pathways. The functions of 2,510 differentially expressed genes were found to be associated with disease occurrence. Furthermore, a network comprising 8 key strains, 14 key genes and 83 key metabolites was constructed.

**Conclusion:**

Through this study, we conducted a comprehensive analysis of NAFLD alterations, exploring the gut microbiota, genes and metabolites of the results offer insights into the speculated biological mechanisms underlying NAFLD.

## 1 Introduction

The gut microbiota (GM) constitutes a complex, dynamic and spatially heterogeneous ecosystem hosting numerous interacting microorganisms (Fan and Pedersen, 2021). Renowned as not only the largest “micro-ecosystem,” but also the “second largest gene pool” in the human body (Ren et al., 2020; Li C. et al., 2021). GM plays a pivotal role in human health (Álvarez-Mercado et al., 2019). Alterations in the GM composition can significantly impact metabolic health, with abnormal changes in its abundance contributing to various common metabolic disorders, such as obesity, type 2 diabetes, non-alcoholic fatty liver disease, and cardiometabolic disease (de la Cuesta-Zuluaga et al., 2018; Safari and Gerard, 2019; Sanchez-Rodriguez et al., 2020; Wu et al., 2020). Marshall proposed the concept of the entero-hepatic axis (Marshall, 1998), elucidating the reciprocal regulation and influence of substances, cells and cytokines between the liver and intestine through the portal vein system. Moreover, studies have validated the metabolic interaction and immune correlation between the liver and intestine.

Studies have shown that in healthy individuals, there is usually a stable proportion of bacteria within the GM. Disruption of the GM can lead to structural, functional and diversity changes in intestinal tissues. Moreover, an increase in pathogenic bacteria could cause inflammation, energy metabolism disorders and immune disorders in host tissues, potentially leading to various diseases (Singh et al., 2017), including non-alcoholic fatty liver disease (NAFLD). According to the latest nomenclature standards, Metabolic Associated Steatotic Liver Disease (MASLD) has replaced the previous term Non-Alcoholic Fatty Liver Disease (NAFLD). However, for the sake of clarity and reference in this study, we will continue to use the term NAFLD after introducing MASLD. MASLD is a newer term. It is defined by hepatic steatosis and the presence of at least one cardio-metabolic risk factor. It emphasizes the central role of metabolic factors in fatty liver disease. This name more clearly points out the key role of metabolic disorders (such as obesity, diabetes, hyperlipidemia, etc.) in the occurrence of disease (Abdelhameed et al., 2024). NAFLD encompasses liver fat accumulation exceeding 5%, excluding alcohol and known liver damage factors, and includes non-alcoholic hepatic steatosis, non-alcoholic steatohepatitis (NASH), cirrhosis, and hepatocellular carcinoma (Powell et al., 2021). Research by Li F. et al. (2021) confirmed increased abundances of *Escherichia*, *Prevotella* and *Streptococcus*, alongside decreased abundances of *Coprococcus*, *Faecalibacterium* and *Ruminococcus* in patients with NAFLD. Similarly, a study by Loomba et al. (2017) also identified GM imbalances in NAFLD, specifically increased abundances of *Proteobacteria* and *Enterobacteria* and decreased abundances of *Ruminococcus* and *Firmicutes*. Further investigations revealed the crucial role of GM metabolites in the onset and progression of NAFLD. However, comprehensive analyses integrating transcriptome-metabolome-and microbiome data remain an area needing further exploration.

The advantage of using mice as a multi-omics model for NAFLD research lies in the following reasons. First, biological similarity. The biology of mice is similar to that of humans, especially in terms of metabolic and liver diseases. Therefore, by studying NAFLD in mouse models, the pathogenesis and pathophysiological processes of the disease can be better understood. Second, controllability. Mouse models can be more easily controlled and manipulated, and experimental conditions such as diet, environment, and gene expression can be precisely controlled to better understand the development and progression of NAFLD. Third, experimental resources and costs. Mouse models are relatively inexpensive and resources are more readily available, and mouse experiments can be conducted more cost-effectively than human studies. Fourth, evaluation of potential treatment strategies. Studies conducted in mouse models can provide important information for the development and evaluation of potential NAFLD treatment strategies, such as drug therapy, dietary interventions, and gene editing.

In this study, we extensively elucidated the intricate biological mechanisms involved in the pathogenesis of NAFLD, examining genes, species, metabolites and metabolic pathways through multi-omics analyses encompassing the microbiome, metabolome and transcriptome. Specifically, we initially employed 16S rRNA sequencing to analyze differences in the species diversity and composition within the GM between patients with NAFLD and healthy controls (HCs). Subsequently, we identified differential microbiota, and metabolites as well as differentially expressed genes (DEGs), exploring their respective functions. Additionally, we delved into the driving mechanisms associated with key strains of NAFLD by conducting correlation analyses among the genes, microbiota and metabolites.

## 2 Materials and methods

### 2.1 Construction of a nonalcoholic fatty liver model

Male C57 mice aged 8 weeks and weighing between 18 and 22 g were randomly allocated into two groups: 9 mice in the high-fat group and 8 in the control group. The high-fat group was fed through 85% basal feed +15% lard +1.5% cholesterol; the low-fat group was fed through normal feed. The feed composition includes 97 g/kg moisture, 194.8 g/kg crude protein, 46 g/kg crude fat, 20 g/kg crude fiber, 56 g/kg crude ash, 13.4 g/kg calcium, 8.2 g/kg total phosphorus, 0.146 mg/kg aflatoxin B1, and < 10 CFU/g total colony count. All mice had *ad libitum* access to food and water throughout the study. The high-fat group received a diet high in -fat content, whereas the control group was provided with a standard normal diet. After a feeding period of 13 weeks, blood samples were collected from the eyeball, fecal samples were collected and liver tissue was obtained through laparotomy. This study received approval from the ethics committee of Kunming Medical University.

### 2.2 Lipid panel tests

The blood collected from the eyeballs in each group at a range of 2–8°C by centrifugation at 3000 rpm for 15 min. Subsequently, the resulting and the supernatant underwent analysis using a biochemical analyzer (SMT-120VP, Smart).

### 2.3 Fat expression detection using oil red O staining

The liver tissues obtained from the mice in each group were initially fixed in paraformaldehyde, and subsequently treated with the OCT embedding agent, and rapidly frozen and embedded on the frozen table of microtome. To visualize lipid content, frozen sections were immersed in an oil red O working solution in darkness for 8–10 min. Afterwards, differentiation was achieved by briefly submerging the sections in 60% isopropyl alcohol for 3–5 s. The staining of nuclei was accomplished using a haematoxylinstaining solution (G1004, Servicebio), followed by a series of washing steps with water to remove excess stain. Once washed, dried slightly, the sections were sealed using a glycerine gelatine sealing medium to preserve the samples.

### 2.4 Data extraction

The total fecal microbiota DNA was extracted and the V3-V4 regions of the 16S rRNA gene were amplified. DNA extraction kits were available for different types of samples in order to ensure DNA extraction efficiency and quality. (OMEGASoilDNAKit; OMEGAWaterDNAKi; OMEGAStool DNAKit). The PCR primer (Supplementary Table 1) was designed against the conserved region to target the variable region of the 16S /ITS2 rDNA gene. After 35 cycles of PCR, sequencing adapters and barcodes were added for amplification. PCR amplification products were detected by 1.5% agarose gel electrophoresis. The target fragments were recovered using the AxyPrep PCR Cleanup Kit. The PCR product was further purified using the Quant-iT PicoGreen dsDNA Assay Kit. The library was quantified on the Promega QuantiFluor fluorescence quantification system. The pooled library was loaded on Illumina platform using a paired-end sequencing protocol (2 × 250 bp). The composition of the fecal microbiota was evaluated using the quantitative insights into microbial ecology (QIIME) software (Release 138) for microbiota analysis.

Additionally, the collected tissue samples were thawed on ice, and metabolites were extracted using a 50% methanol buffer. Briefly, 20 μL of each sample was mixed with 120 μL of precooled 50% methanol, vortexed for 1 min, and then incubated at room temperature for 10 min. The extraction mixture was stored overnight at −20°C. The next day, the mixture was centrifuged at 4,000 *g* for 20 min, and the supernatants were carefully transferred into new 96-well plates. The samples were then stored at −80°C until further analysis by LC–MS. In addition, quality control (QC) samples were prepared by combining 10 μL of each extraction mixture. QC samples with a relative standard deviation (RSD) greater than 30% were removed from the analysis to ensure data quality. Subsequently, the extracted metabolites were annotated and quantified using the metaX software in combination with the KEGG and HMDB databases to identify and characterize the metabolites of interest.

### 2.5 Species diversity analysis

The sequencing depth was assessed using the Good’s coverage index. Various a Alpha-diversity indexes, including Chao1, observed species, Shannon and Simpson, were compared to analyze the species richness and uniformity (Mohakud et al., 2019). Beta-diversity analysis was used to evaluate the species’ complexity among different groups. In this study, principal coordinate analysis was used to illustrate the differences between the different groups. Subsequently, the analysis of similarities (Anosim) was used to analyze whether differences between the two groups were significantly greater than those within the groups (Draiko et al., 2019).

### 2.6 Species composition diversity analysis

The GM composition was further analyzed, comparing the distribution of the GM at both the phylum and genus levels. Characteristic strains were identified using linear discriminant analysis effect size (LEfSE), and operational taxonomic units with a relative abundance exceeding 0.5% were selected for analysis based on the linear discriminant analysis (LDA) scores (Segata et al., 2011).

### 2.7 Functional prediction for the diagnostic strains

Receiver operating characteristic (ROC) curves were calculated using the “pROC” R package (version 1.18.0), and the area under the ROC curve (AUC) values were used to access the distinguish ability of characteristic strains (Robin et al., 2011). Functional prediction of the diagnostic strains (AUC > 0.8) was assessed, and differences between NAFLD mice and HCs were studied using the “PICRUSt2” R package (version 0.2.3) (Douglas et al., 2020).

### 2.8 Differential analysis of the metabolites

The metabolic differences between the NAFLD mice and HCs were compared through the construction of an orthogonal partial least squares discriminant analysis (OPLS-DA) model. This model’s validity was assessed using a permutation test. Subsequently, three methods, were employed to select metabolites: the multiple change method (FC value), the t test method (*p* value, and FDR value), and the partial least squares discriminant analysis method (VIP value). The differentially expressed metabolites between the two groups were identified using the following criteria: VIP > 1, FC ≥ 1, and *p* < 0.05. In addition, enrichment analysis of these differentially expressed metabolites was performed using the online platform “Metaboanalyst” (Chong et al., 2019).

### 2.9 Function analysis of the DEGs in NAFLD

The mRNA expression levels between the NAFLD mice and HCs were compared using the “limma” R package (version 3.52.4) (|logFC| > 0.5, *p* < 0.05), and visualized using the ‘ggplot2’ R package and Kolde R (2019). _pheatmap: Pretty Heatmaps_. R package version 1.0.12.^1^ Then, Gene Ontology (GO) and Kyoto Encyclopedia of Genes and Genomes (KEGG) enrichment analyses of DEGs were conducted using the ‘clusterprofiler’ R package (version 4.4.4) (Wu et al., 2021).

### 2.10 Construction of a gene-microbiota-metabolite interaction network

The correlations between the characteristic strains and differential metabolites were assessed using the ‘Spearman’ package (|r| ≥ 0.3, *p* < 0.05). Metabolites in the pair were regarded as the key metabolites 1, and diagnostic strains were regarded as key microbiota. Similarly, the correlations between the DEGs and differential metabolites were studied, and the DEGs in the pair were regarded as key genes, whereas metabolites were regarded as key metabolites 2. Collinear networks of the key microbiota-metabolites 1 and key genes-metabolites 2 were constructed using ‘Cytoscape’ (version 3.7.1) (Shannon et al., 2003). Finally, key metabolites 1 and 2 were combined, and a collinear network of key genes-microbiota-metabolites was constructed using ‘Cytoscape.’

### 2.11 Verification of the expression of differential genes using RT-PCR

Total RNA was extracted from the control and model groups, and the reverse transcription reaction was subsequently performed, followed by qPCR reaction with 2 × Universal Blue SYBR Green qPCR Master Mix kit. The PCR cycling conditions were as follows: pre-denaturation at 95°C for 1 min; denaturation for 20 s at 95°C, annealing for 20 s at 55°C,and final extension for 30 s at 72°C; total, 40 cycles.

### 2.12 Statistical analysis

Statistical analysis was performed using R software (version 4.2.3) and the maps were plotted using Prism GraphPad, and the experimental data are presented as mean ± SEM. The Welch’s *t*-test was used to compare the measurement data between groups. *p* < 0.05 for statistical significance; *p* < 0.01 for high statistical significance; *p* < 0.001 for extremely high statistical significance.

## 3 Results

### 3.1 Construction of non-alcoholic fatty liver model

**Figure 1:**
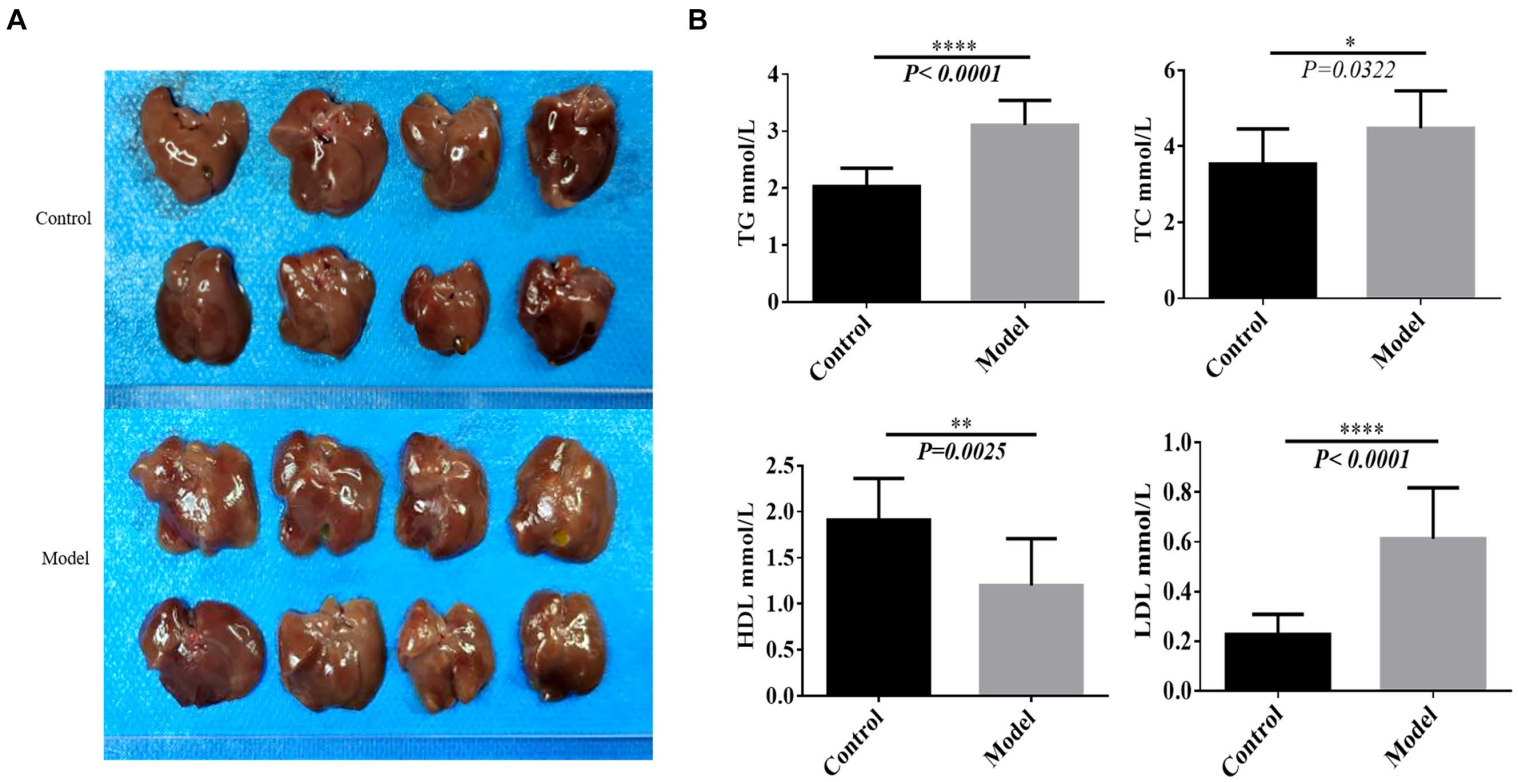
Construction of the nonalcoholic fatty liver model. **(A)** The liver sample of each tissue. **(B)** Lipid panel. *p < 0.05, **p < 0.01, ****p < 0.0001.

Upon examination, liver tissues from the model group of C57 mice exhibited a distinctive yellow hue and enlarged sizes (Figure 1A). Moreover, the levels of TG, TC and LDL were significantly increased, whereas HDL levels showed a significant decrease (Figure 1B). These findings collectively suggest the successful establishment of the NAFLD model (Figure 1).

### 3.2 Fat formation observed upon oil red O staining

**Figure 2:**
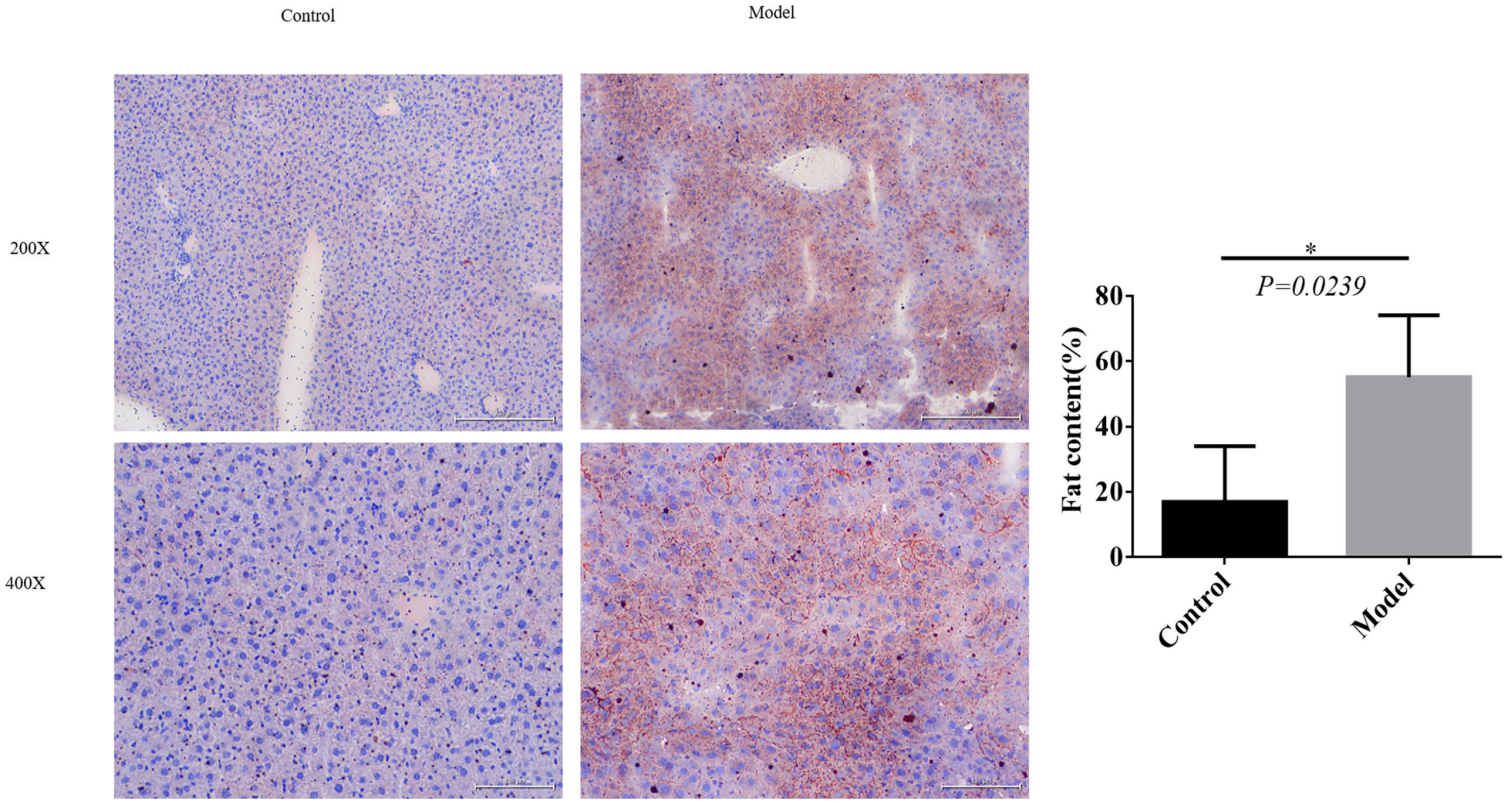
Fat formation observed upon oil red O staining. *p < 0.05.

Excessive fat deposition represents a crucial pathological characteristic of NAFLD. To validate the successful establishment of the model, we conducted observations of adipogenesis using oil red O staining. Results demonstrated a substantial increase in fat content within the model group compared to the control group, further substantiating the successful construction of the model (Figure 2).

### 3.3 Species diversity analysis

**Figure 3:**
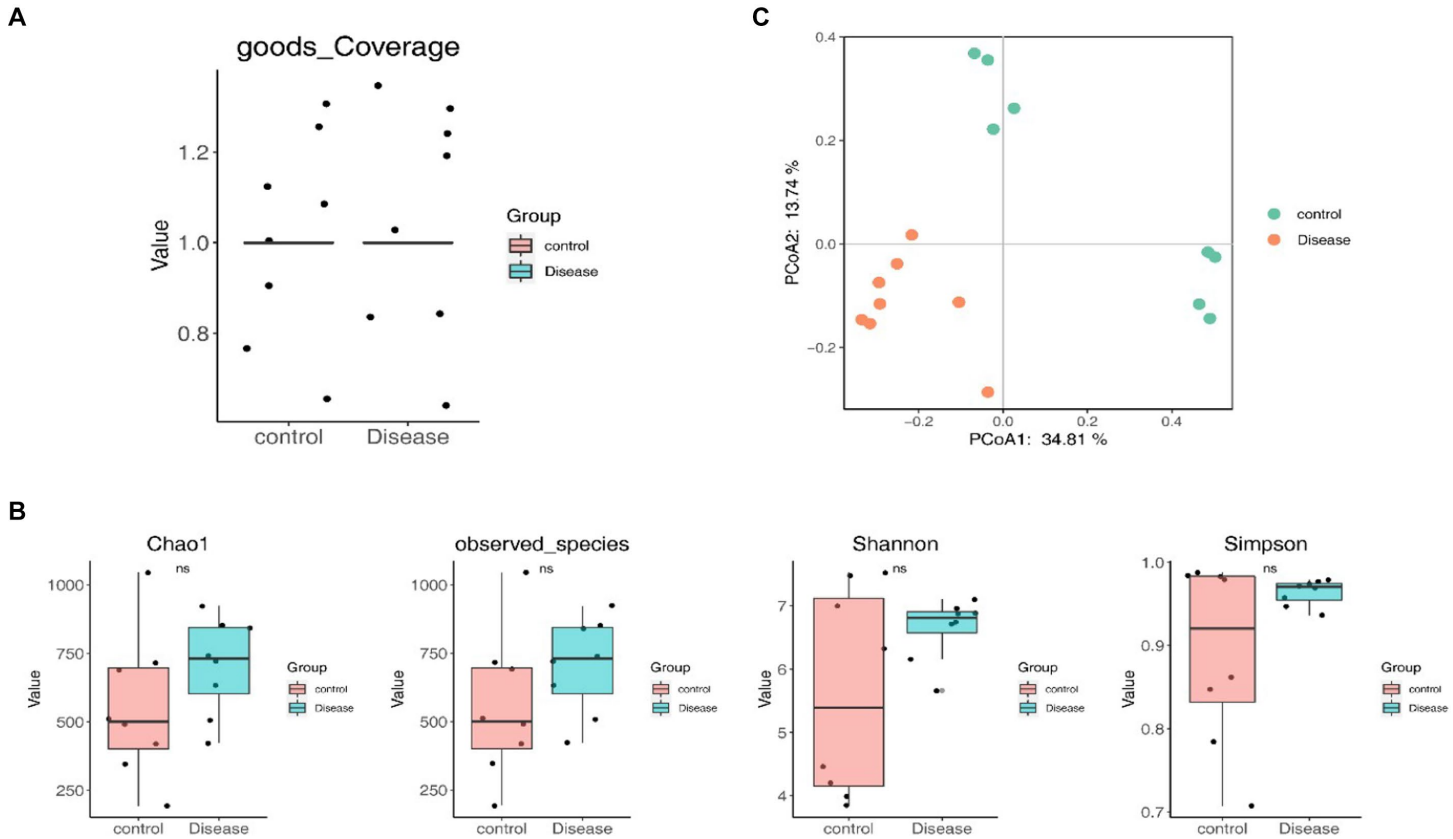
Species diversity analysis. **(A)** The goods coverage curve. **(B)** Alpha-diversity indexes between the NAFLD and HC groups. **(C)** The PCoA result.

The goods coverage curve approaching 1, indicated that sufficient sequencing data were acquired, affirming a reasonable sequencing depth (Figure 3A). No significant differences in alpha-diversity indexes were observed between the NAFLD and HC groups, suggesting that the richness and uniformity of bacterial communities remained largely unchanged owing to NAFLD (Figure 3B). However, the PCoA results showed a distinct clustering of patients with NAFLD and HCs (Figure 3C). Furthermore, the ANOSIM analysis demonstrated greater differences between groups than within groups (ANOSIM statistics = 0.6233, *p* = 0.001), signifying the meaningfulness of grouping and substantial alterations in the structure and composition of the GM induced by NAFLD.

### 3.4 Analysis of the species composition diversity

**Figure 4:**
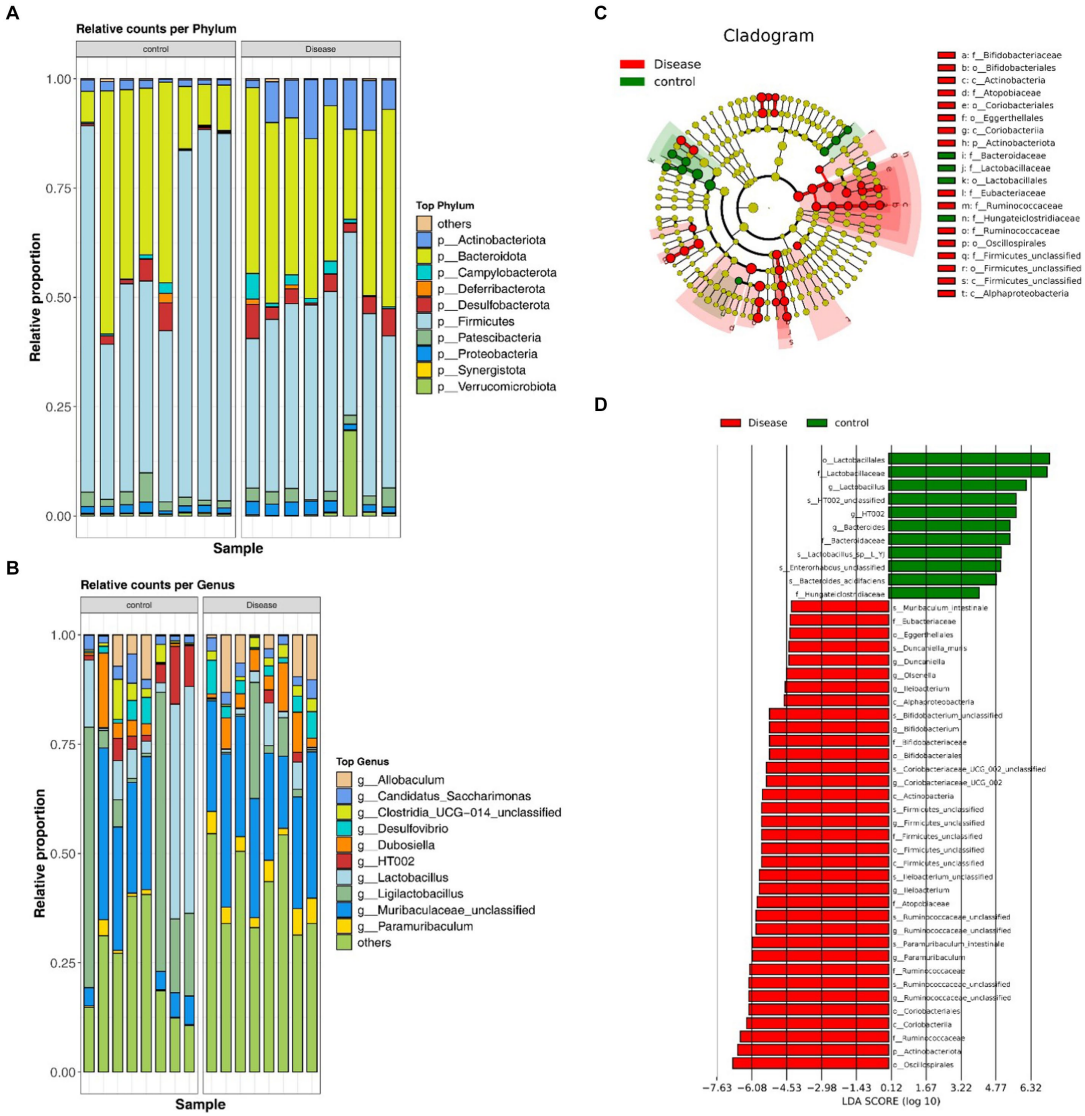
Analysis of the species composition diversity. **(A)** The distribution of the GM at the phylum level. **(B)** The distribution of the GM at the genus level. **(C)** LEfSE analysis of the clustering tree. **(D)** Different microflora in the NAFLD and HC groups.

At the phylum level, the gut microbiota primarily comprised Firmicutes, Bacteroidetes, Actinobacteria, Desulfobacterota, Patescibacteria, Proteobacteria, Verrucomicrobiota, Campylobacterota, Deferribacterota, and Synergistota. Comparing the NAFLD and HC groups, revealed significant decreases in the abundances of Firmicutes and Synergistota in the NAFLD groups (*p* < 0.05), whereas Patescibacteria and Deferribacterota showed decreased abundance, Others demonstrated significantly decreased abundances in the NAFLD group (*p* < 0.05) (Figure 4A and Supplementary Table 2). At the genus level, the NAFLD group exhibited prominent representation of *Muribaculaceae, Allobaculum, Dubosiella, Ligilactobacillus, Ruminococcaceae, Paramuribaculum, Bifidobacterium*, and *Desulfovibrio*, among others. The HC group showed a predominant presence of *Ligilactobacillus, Muribaculaceae, Lactobacillus, HT002, Dubosiella, Allobaculum, Candidatus*, and *Clostridia*, among others. Notably, the NAFLD group demonstrated significantly increased abundances of *Muribaculaceae, Allobaculum, Paramuribaculum*, and *Ruminococcaceae*, and decreased abundances of *Ligilactobacillus, Lactobacillus*, and *HT002* (Figure 4B and Supplementary Table 3). Further investigation using LEfSE analysis identified 46 differentially abundant key gut microbiota, including 35 with notable roles in the NAFLD groups and 11 in the HC groups. Among these, *Ileibacterium, Ruminococcaceae* (Firmicutes), *Olsenella, Duncaniella* and *Paramuribaculum* (Bacteroidota), and *Bifidobacterium, Coriobacteriaceae_UCG_002* and *Olsenella* (Actinobacteriota) were characteristic strains of NAFLD. On the other hand, *HT002, Lactobacillus* (Firmicutes), *Bacteroides* (Bacteroidota) were characteristic strains of HC (Figures 4C,D and Supplementary Table 4).

### 3.5 Screening of the 13 diagnostic strains, associated with liver protection

**Figure 5:**
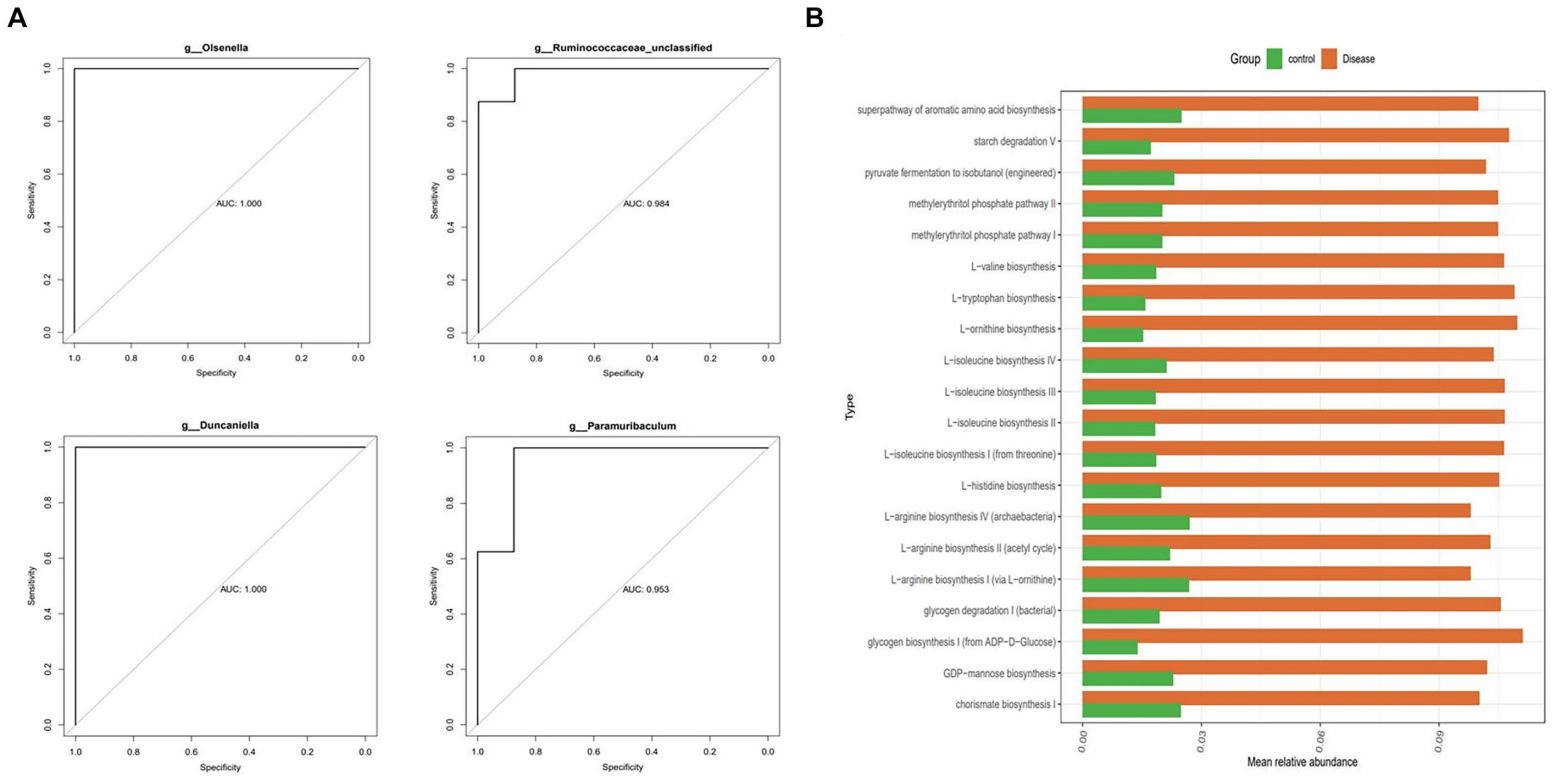
The 13 diagnostic strains related to liver protection and their function prediction analysis. **(A)** The ROC curve characteristic pressure calculation and 4/13 diagnostic strains produced AUC values >0.8. **(B)** Functional prediction analysis of diagnostic strains.

In this study, the ROC curves of the characteristic strains were calculated, and 13 diagnostic strains were obtained with an AUC value >0.8 (AUC*_Bifidobacterium_* = 0.969, AUC*_Olsenella_* = 1.000, AUC*_Enterorhabdus_* = 0.812, AUC*_Bacteroides_* = 0.812, AUC*_Duncaniella_* = 1.000, AUC*_Muribaculum_* = 0.922, AUC*_Paramuribaculum_* = 0.953, AUC*_Ileibacterium_* = 0.891, AUC*_Turicibacter_* = 0.922, AUC*_HT002_* = 0.883, AUC*_Lactobacillus_* = 0.979, AUC*_Ruminococcaceae_* = 0.984, AUC*_Ileibacterium_* = 0.891) (Figure 5A). In addition, the functional prediction of the diagnostic strains was assessed, and the results showed that 121 pathways were significantly different between the two groups. Among these groups, methylerythritol phosphate pathway, glycogen degradation, l-ornithine, l-valine, l-tryptophan, and l-isoleucine biosynthesis pathways, among others, were significantly highly expressed in the NAFLD groups (*p* < 0.05) (Figure 5B).

### 3.6 The function of 2,497 differential metabolites mainly enriched in tryptophan, linoleic acid and methylhistidine metabolism pathways

**Figure 6:**
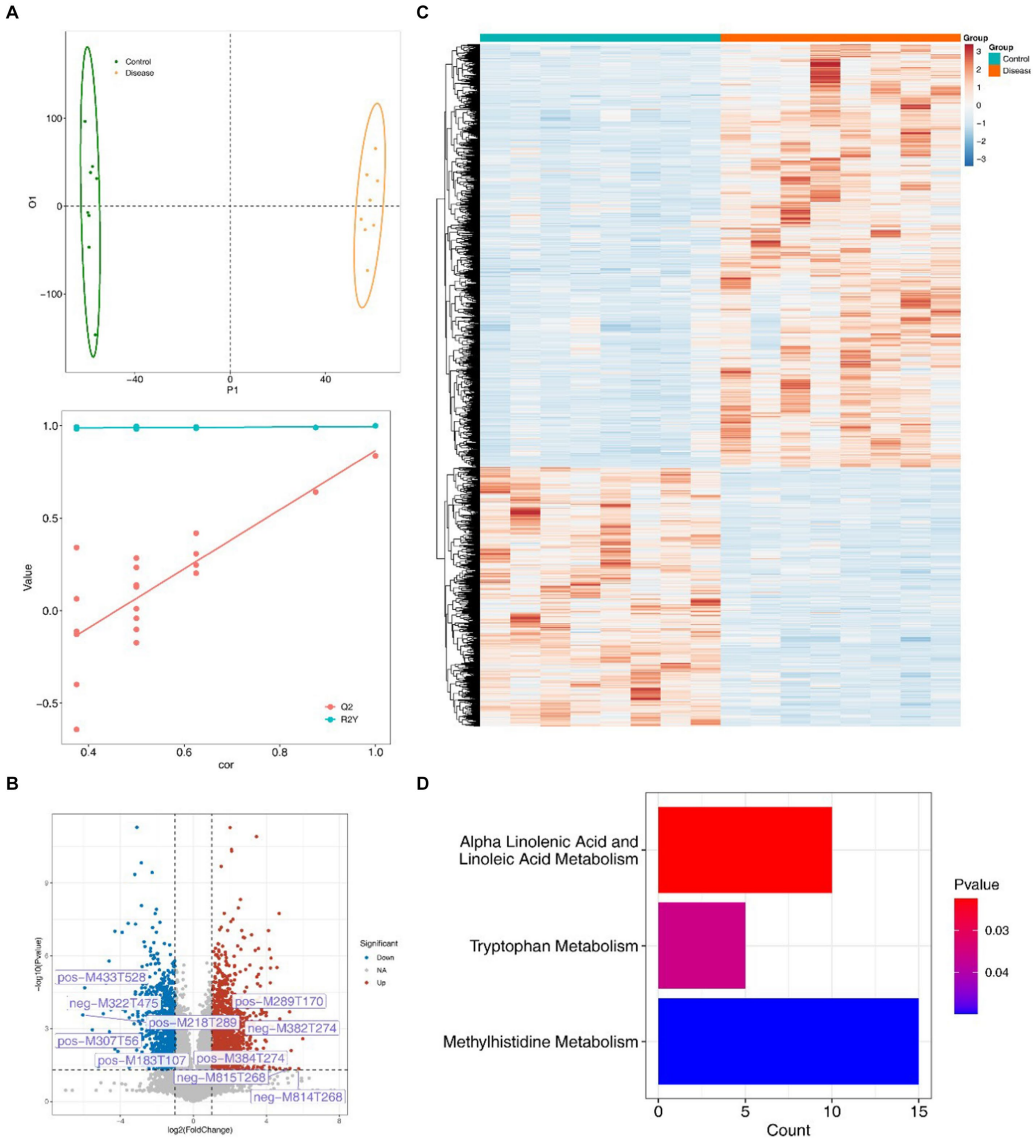
2,497 different metabolites and their functional enrichment metabolic pathways. **(A)** The OPLS-DA model shows differences in the metabolite composition between the two groups. **(B)** The volcanic map of different metabolites in the NAFLD group with VIP > 1, FC ≥ 1, *p* < 0.05. **(C)** Heat map of differential metabolite expression in the NAFLD group. **(D)** Differential metabolite enrichment pathway.

The constructed OPLS-DA model revealed distinct differences in metabolite composition between the two groups (*Q*2 = 0.837, R2Y = 0.999) (Figure 6A). A total of 2,497 differentially regulated metabolites, including 947 down-regulated and 1,551 up-regulated metabolites, were identified in the NAFLD group, meeting the criteria of VIP > 1, FC ≥ 1, and *p* < 0.05 (Figures 6B,C). In addition, these differentially regulated metabolites were predominantly enriched in pathways related to tryptophan, alpha-linolenic acid, linoleic acid and methylhistidine metabolism (Figure 6D).

### 3.7 The functions of 2,510 DEGs are associated with the occurrence of diseases

**Figure 7:**
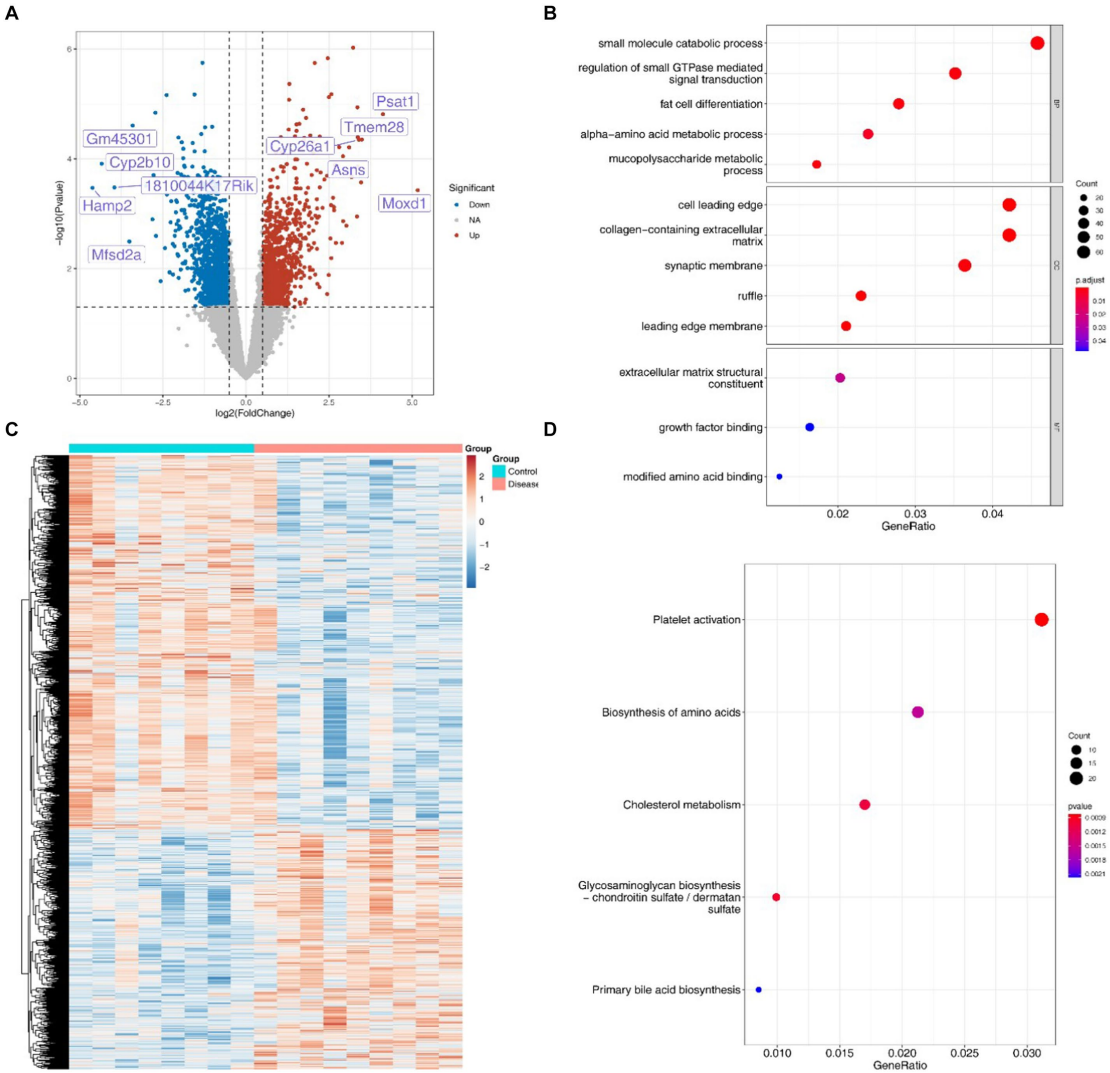
Differentially expressed genes and their enrichment analysis. **(A)** Volcanic map of 2,510 DEGs associated with disease occurrence. **(B)** Heat maps of 2,510 DEGs associated with disease occurrence. **(C)** GO enrichment analysis of DEGs. **(D)** KEGG enrichment analysis of DEGs.

A total of 2,510 DEGs (1,531 down-regulated and 979 up-regulated) were screened in the NAFLD groups compared with the HC groups (Figures 7A,B). In addition, these DEGs exhibited significant enrichment in 92 GO, categories, notably involving processes such as small molecule catabolic, processes and the regulation of small GTPase mediated signal transduction (Figure 7C). Furthermore, the KEGG results revealed associations of these DEGs with pathways related to cholesterol metabolism, amino acid biosynthesis, and primary bile acid biosynthesis, among others (Figure 7D).

### 3.8 Correlation analysis among the genes, microbiota and metabolites

**Figure 8:**
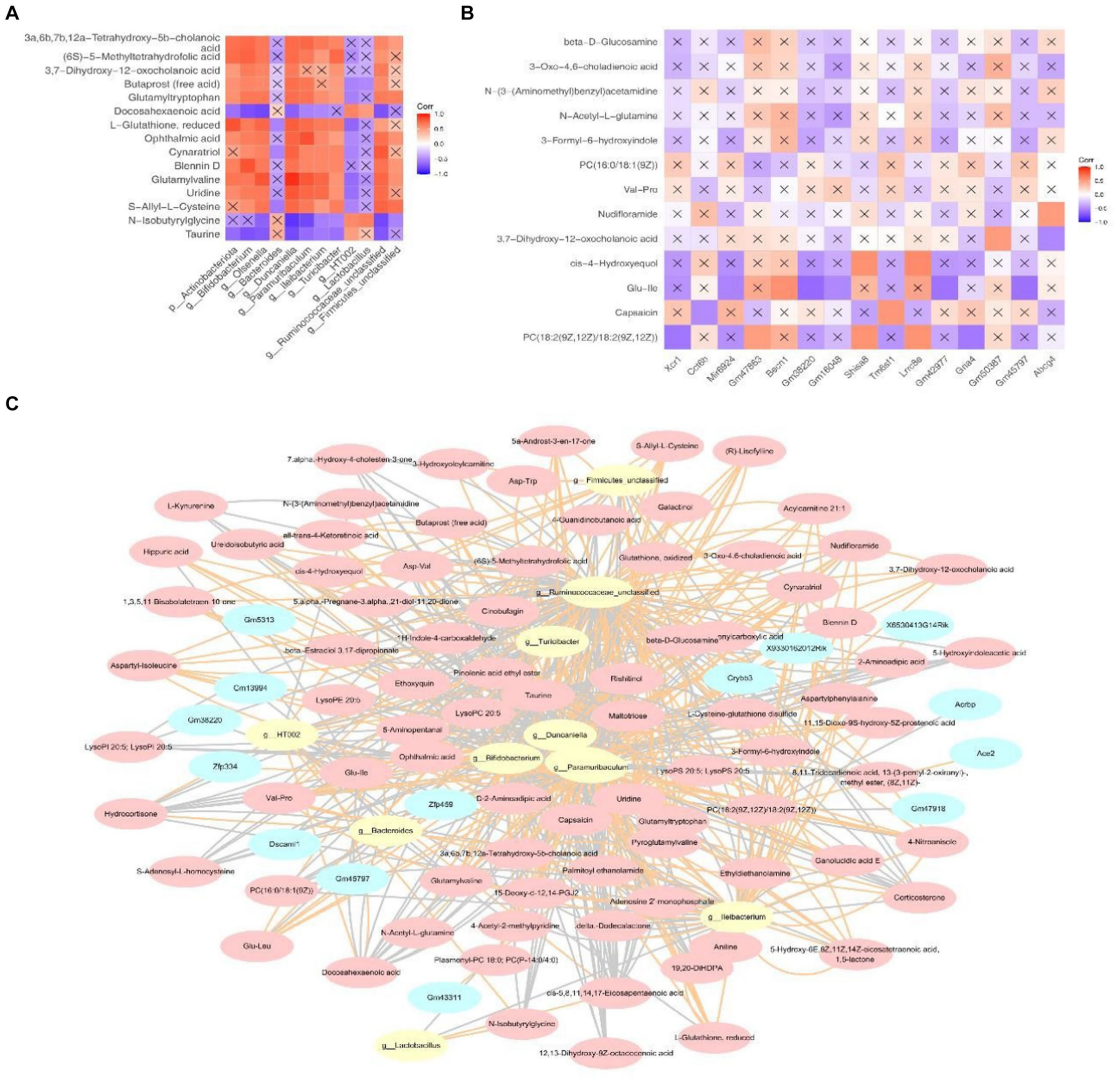
The correlation between the key metabolites, microbiota and DEGs and the collinear network diagram. **(A)** Correlation between the key metabolites and microbiota. **(B)** Correlations between some differential metabolites and DEGs. In Figures, the “X” indicates non-significant (*p* > 0.05). **(C)** Collinear network diagram of the genes –microbiome –metabolites.

As shown in Figure 8A, characteristic strains of the HC groups—*Bacteroides, HT002* and *Lactobacillus* demonstrate strong negative correlations with docosahexaenoic acid, n-isobutyrylglycine and taurine. Conversely, the key microbiota of the NAFLD groups exhibit strong positive correlations with cholanoic acid, 5-methyltetrahydrofolic acid etc. The correlation between the key metabolites 2 and genes is shown in Figure 8B. Shisa8 and Lrrc8e display strong positive correlations with cis-4-hydroxyequol, whereas Gm38220 and Gm42977 exhibit strong negative correlations with Glu-Ile. Moreover, the constructed network involving 8 key strains, 14 key genes and 83 key metabolites elucidates potential regulatory interactions. Notably, Mup-ps16 has been indicated to potentially regulate the metabolism of 3,7-dihydroxy-12-oxocholanoic acid through interactions with *Duncaniella* and *Olsenella*. Additionally, Proser2 shows potential regulatory capabilities across the metabolism influenced by most diagnostic microbiota (Figure 8C and Supplementary Table 5).

### 3.9 Verification of the expression of differential genes using RT-PCR

**Figure 9:**
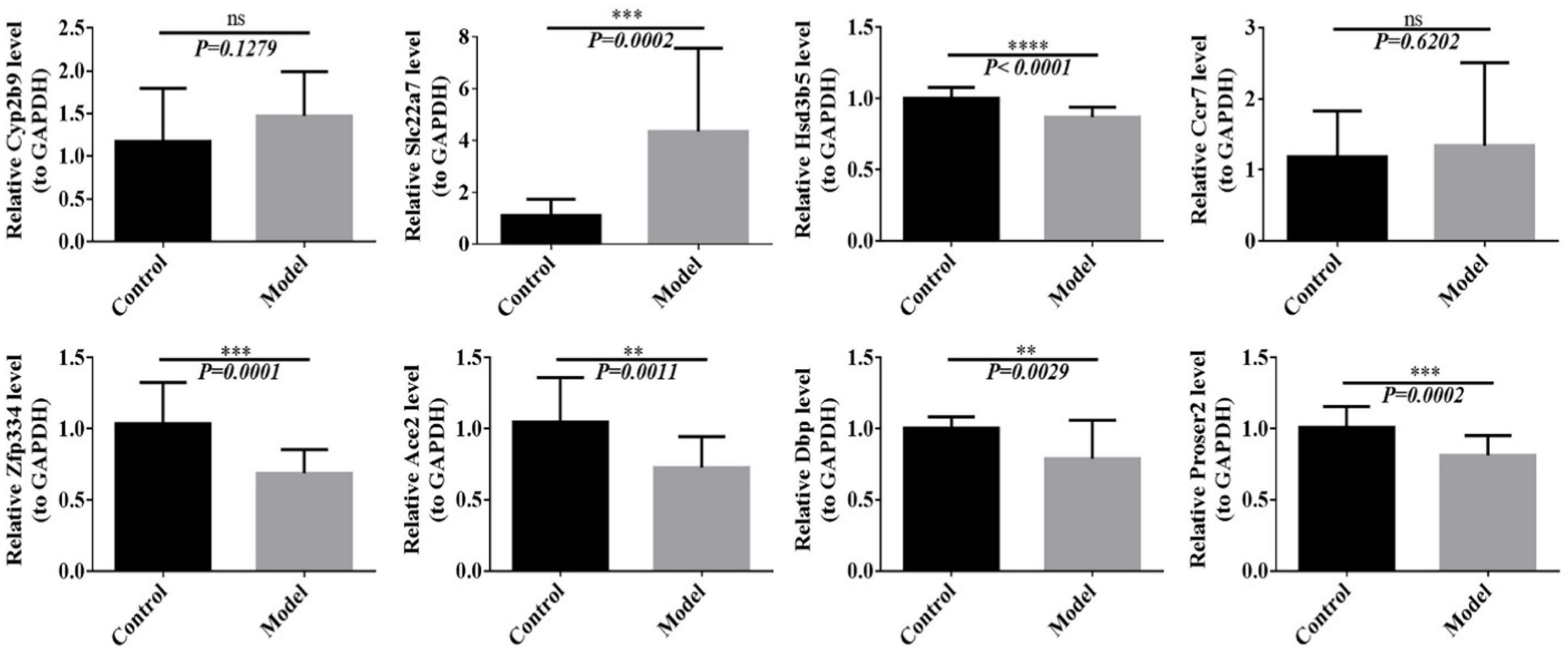
The expression of differential genes was detected by RT-PCR. **p < 0.01, ***p < 0.001, ****p < 0.0001.

Based on the results of multi-omics joint analyses, we further verified the expression of differential genes using RT-PCR. The results showed that compared with that in the control group, the expression of Slc22a7 in the model group was significantly increased, whereas the expressions of Hsd3b5, Zfp334, Ace2, Dbp and Proser2 were significantly decreased. Cyp2b9 and Ccr7 showed no significant differences. In addition to Dbp, Hsd3b5, Cyp2b9 and Ccr7, Slc22a7, Zfp334, Ace2 and Proser2 expressions were consistent with the results of multi-omics analysis (Figure 9).

## 4 Discussion

With the advancement in people’s living standards, NAFLD has emerged as the most prevalent liver disease globally. An increasing body of research has established connections between imbalances in gut flora and a spectrum of host diseases, such as fatty liver disease, metabolic syndrome, inflammatory bowel disease, and colon cancer. The direct linkage between the intestine and liver through the portal vein, enables the intestinal microflora to remotely influence liver health (gut–intestinal microbiota – liver axis). Consequently, disruptions in intestinal microflora can alter the biological metabolism beyond the liver, potentially contributing to the onset and development of NAFLD. However, comprehensive joint analyses integrating transcriptomic, microbiomic and metabolomic data in NAFLD remain unreported.

In this study, we observed significant increases in the abundances of *Bacteroides*, *Muribaculaceae, Allobaculum, Paramuribaculum* and *Ruminococcaceae*, alongside notable decreases in the abundances of *Ligilactobacillus, Lactobacillus* and *Enterococcus faecalis HT002* within the NAFLD group. Specifically, *Ileibacterium valens, Ruminococcaceae, Duncaniella frettoniella, Paramuribaculum, Coriobacteriaceae, Marmorella UCG_002* and *Olsenella* were identified as characteristic strains associated with NAFLD. Previous studies have highlighted the negative association of the *Ruminococcaceae* family with hepatic markers, including liver weight, serum transaminase levels and the degree of hepatic steatosis and inflammation (Milton-Laskibar et al., 2022). Moreover, our findings align with previous reports indicating increased abundances of *Blautia, Romboutsia, Faecalibaculum* and *Ileibacterium* alongside decreased levels of *Allobaculum* and *Enterorhabdus* in NAFLD mice (Gu et al., 2022). Our results are consistent with those of previous literature reports. The relationship between the other five strains and NAFLD has not been reported in detail. However, Fang et al. (2022) found that *Olsenella* is negatively correlated with lower levels of free fatty acids, a pivotal factor in alcoholic liver disease- development. Additionally, Zhou et al. (2022) investigated the intestinal microecology in hepatocellular carcinoma model mice induced by diethylnitrosamine, emphasizing *Coriobacteriaceae* as a dominant bacterial group at the genus level. This further suggests the potential importance of *Olsenella* and *Coriobacteriaceae* in liver diseases.

Jiang et al. (2023) discovered that free fatty acid receptor4 (FFAR4) deficiency hindered the protective effects of high endogenous n-3 polyunsaturated fatty acids on intestinal barrier dysfunction and hepatic steatosis. Moreover, FFAR4 deficiency decreased gut microbiota diversity and increased the prevalence of *Rikenella, Anaerotruncus*, and *Enterococcus*, while reducing that of *Dubosiella*, *Ruminococcaceae UCG-010, Ruminococcaceae UCG-014, Coriobacteriaceae UCG-002, Faecalibaculum, Ruminococcaceae UCG-009*, and *Akkermansia*. Alterations in the abundances of these specific bacterial genera led to changes in the overall production of free fatty acids and their interaction manner with FFAR4, ultimately diminishing the protective mechanisms against hepatic steatosis. These findings underscore the potential significance of *Ruminococcaceae* and *Coriobacteriaceae* in liver diseases.

We observed significantly higher expression levels of the methylerythritol phosphate, glycogen degradation, l-ornithine, l-valine, l-tryptophan, and l-isoleucine biosynthesis pathways in the NAFLD group. Isoprenoids were associated with the methylerythritol phosphate pathway. Glycogen degradation was found to be correlated with liver metabolism, processes, contributing to the onset and development of NAFLD. L-ornithine and L-aspartic acid (LOLA) act as effective amino-decreasing agents in hepatic encephalopathy (Teunis et al., 2022). LOLA facilitates ammonia removal through urea synthesis and glutamine production via the action of glutamine synthetase. Plausible mechanisms of LOLA in NAFLD involve enhanced ammonia removal, increased antioxidative capacity, and reduced lipid peroxidation via the action of glutamine and glutathione. Additionally, LOLA improves hepatic microcirculation by producing l-arginine-derived NO (Canbay and Sowa, 2019).

Furthermore, we identified key metabolites significantly enriched in tryptophan metabolism, alpha-linolenic acid linoleic acid metabolism and methylhistidine pathways. Notably, NAFLD, a known risk factor for cirrhosis, is linked with metabolic conditions such as obesity, type 2 diabetes, dyslipidaemia, and atherosclerosis, conditions potentially mitigated by tryptophan metabolism. The canine pathway, a significant route in tryptophan metabolism is modulated by indoleamine 2, 3-dioxygenase, whose increased activity is positively associated with inflammation and fibrosis in NAFLD (Teunis et al., 2022). In addition, substituting linoleic acid or long-chain n-3 polyunsaturated fatty acids has shown potential in preventing the onset of NAFLD associated with a western diet (Musso et al., 2010; Yuan et al., 2016).

The key microflora associated with NAFLD, such as *Muribaculaceae, Allobaculum, Dubosiella, Ligilactobacillus, Ruminococcaceae, Paramuribaculum*, and *Bifidobacterium*, exhibited strong positive correlations with cholinic acid, 5-methyltetrahydrofolic acid and other substances. A study has indicated that primary bile acids and deoxycholic acids tend to accumulate in mice on a choline-deficient diet. Moreover, the introduction of *Aerococcus, Oscillospiraceae, Ruminococcaceae, Bilophila, Muribaculaceae, Helicobacter* and *Alistipes* has been linked to increased liver steatosis, lobular inflammation and fibrogenesis in mice, consequently promoting the progression of NAFLD (Jian et al., 2022). These findings align with our analyses.

In this study, our analysis revealed associations of DEGs with processes such as small molecule catabolism and the regulation of small GTPase-mediated signal transduction. Notably, NAFLD can progress into NASH, where the activation of the inflammasome protein scaffold (NLRP3) plays a pivotal role in NASH-related inflammation. Moreover, our findings indicate that Mup-ps16 could regulate the metabolism of 3,7-dihydroxy-12-oxocholanoic acid through the action of *Duncaniella* and *Olsenella*. Similarly, Proser2 appears to regulate metabolism across most diagnostic microbiota. Mouse major urinary proteins, primarily expressed in the liver, and circulating major urinary proteins regulate metabolism by suppressing hepatic gluconeogenesis and lipid metabolism (Charkoftaki et al., 2019). In line with our observations, Xiang et al. (2021) suggested that *Olsenella* and *Slackia* genera potentially contribute positively to fat and energy metabolism, correlating with increased chicken abdominal fat deposition. Based on these findings, we hypothesize that Mup-ps16 and *Duncaniella* may regulate the production of 3, 7-dihydroxy-12-oxocholanoic acid, impacting energy metabolism by inhibiting liver gluconeogenesis and lipid metabolism, potentially affecting the progression of non-alcoholic fatty liver.

In summary, *Ileibacterium valens, Ruminococcaceae, Olsenella, Duncaniella Frettoniella, Paramuribaculum, Coriobacteriaceae*, and *Marmorella UCG_002* were identified as the characteristic strains associated with NAFLD. Additionally, significant upregulation of pathways such as the methylerythritol phosphate, glycogen degradation, l-ornithine, l-valine, l-tryptophan, and l-isoleucine biosynthesis pathways was observed in the NAFLD group.

Furthermore, our findings suggest that Mup-ps16 might influence the metabolism of 3,7-dihydroxy-12-oxocholanoic acid through the action of *Duncaniella* and *Olsenella*. Similarly, Proser2 seems to regulate metabolism across most diagnostic microbiota. Based on these observations, we hypothesize that Mup-ps16 and *Duncaniella* could potentially regulate the production of 3, 7-dihydroxy-12-oxocholanoic acid, influencing energy metabolism by inhibiting liver gluconeogenesis and lipid metabolism, thereby affecting the progression of non-alcoholic fatty liver.

In the future, we will focus on conducting an in-depth study of the combined mechanisms of gene–strain metabolic processes in NAFLD.

## Data availability statement

The datasets generated/analyzed for this study can be found in the SRA database https://www.ncbi.nlm.nih.gov/sra/PRJNA1068522 after publication/on 2026-03-01.

## Ethics statement

The studies involving humans were approved by the Ethics Committee of Project Application of the First Affiliated Hospital of Kunming Medical University (July 4th, 2023). The studies were conducted in accordance with the local legislation and institutional requirements. Written informed consent for participation was not required from the participants or the participants’ legal guardians/next of kin in accordance with the national legislation and institutional requirements. The animal study was approved by the Ethics Committee of Kunming Medical University (kmmu20231209). The study was conducted in accordance with the local legislation and institutional requirements.

## Author contributions

JL: Conceptualization, Data curation, Formal analysis, Writing – original draft, Project administration, Software, Writing – review & editing. RZ: Conceptualization, Methodology, Validation, Writing – original draft. HL: Investigation, Supervision, Writing – review & editing. YZ: Formal analysis, Funding acquisition, Methodology, Writing – original draft. ND: Writing – original draft, Investigation, Resources. QQ: Writing – review & editing, Formal analysis, Resources. HB: Writing – review & editing, Project administration, Supervision, Visualization. LZ: Writing – review & editing, Project administration, Supervision, Validation. OL: Writing – original draft, Methodology, Software, Visualization. LS: Writing – original draft, Formal analysis, Software. MM: Writing – original draft, Investigation, Visualization. JY: Funding acquisition, Project administration, Writing – review & editing, Supervision.

## Footnotes

^1^ https://CRAN.R-project.org/package=pheatmap
