# Supplementary material for: Multi-omics analysis of the biological mechanism of the pathogenesis of non-alcoholic fatty liver disease
Source: Front Microbiol. 2024 Jul 26;15:1379064. doi: 10.3389/fmicb.2024.1379064 (PMC11310135; doi:10.3389/fmicb.2024.1379064)
Supplement: Supplementary file 1 [file Table_1.docx]

| Primer | Name | Sequence |
| --- | --- | --- |
| Forward Primer | 341 F | 5’-CCTACGGGNGGCWGCAG-3’ |
| Reverse Primer | 805 R | 5’-GACTACHVGGGTATCTAATCC-3’ |

The sequence of Primer
